# SARS-CoV-2 Seroprevalence in Lithuania: Results of National Population Survey

**DOI:** 10.15388/Amed.2020.28.1.2

**Published:** 2021-01-18

**Authors:** Kastytis Šmigelskas, Kęstutis Petrikonis, Vytautas Kasiulevičius, Ramunė Kalėdienė, Audronė Jakaitienė, Snieguolė Kaselienė, Skirmantė Sauliūnė, Aušra Beržanskytė, Mindaugas Stankūnas

**Affiliations:** Health Research Institute, Faculty of Public Health, Lithuanian University of Health Sciences, Kaunas, Lithuania; Department of Neurology, Faculty of Medicine, Lithuanian University of Health Sciences, Kaunas, Lithuania; Faculty of Medicine, Vilnius University, Vilnius, Lithuania; Department of Health Management, Faculty of Public Health, Lithuanian University of Health Sciences, Kaunas, Lithuania; Institute of Data Science and Digital Technologies, Vilnius University, Vilnius, Lithuania; Department of Health Management, Faculty of Public Health, Lithuanian University of Health Sciences, Kaunas, Lithuania; Department of Health Management, Faculty of Public Health, Lithuanian University of Health Sciences, Kaunas, Lithuania; Department of Public Health, Faculty of Medicine, Vilnius University, Vilnius, Lithuania; Department of Health Management, Faculty of Public Health, Lithuanian University of Health Sciences, Kaunas, Lithuania

**Keywords:** Seroepidemiologic studies, SARS-CoV-2, Asymptomatic cases, Lithuania

## Abstract

**Summary. Background.:**

Betacoronavirus SARS-CoV-2 has spread in early 2020 worldwide just in several months. The official statistics are consistently collected, but this is mainly based on symptomatic reports. This study was aimed to estimate the seroprevalence of SARS-CoV-2 infection in Lithuanian population.

**Materials and methods.:**

Study was conducted during August–September 2020 in 6 municipalities of Lithuania. The sample comprised 3087 adult participants from the general population (mean age 53.7 years, 64% female). SARS-CoV-2 antibodies were assessed using AMP IgM/IgG Rapid Test, other data were based on self-report. Seroprevalence was assessed as a crude estimate and as adjusted by sensitivity-specificity of the test.

**Results.:**

The crude seroprevalence in the total sample was 1.9%, the adjusted – 1.4%, ranging from 0.8% to 2.4% across municipalities. Among seroprevalent cases, 67.2% had IgG, 29.3% had IgM, and 3.5% had both IgG and IgM. An increased risk for seropositive test was observed among people who reported having had close contacts with SARS-CoV-2 positives (OR=5.49, p<0.001). At the borderline significance were female gender (OR=1.75, p=0.082) and non-smoking status (OR=2.95, p=0.072). Among the seropositive participants, 69.0% reported having had no COVID-19 symptoms since 1 March 2020, while 31.0% reported having had at least one of the symptoms.

**Conclusions.:**

The SARS-CoV-2 seroprevalence in Lithuanian sample in August–September 2020 was 1.4%, ranging from 0.8% to 2.4% across municipalities. Given the overall official data, by the end of study (11 September 2020) the total COVID-19 rate in Lithuania was 117.5 per 100,000 population or 0.12%. This suggests more than 10 times higher prevalence of virus across the population than the official estimates.

## Abstract (lt)

**SARS-COV-2 serologinis paplitimas Lietuvoje: nacionalinio populiacijos tyrimo rezultatai:**

**Santrauka. Įvadas.**

Beta koronavirusas SARS-CoV-2 išplito pasauliniu mastu 2020 m. pradžioje vos per keletą mėnesių. Oficiali statistika yra renkama sistemingai, tačiau ji labiau remiasi simptominiais atvejais. Šis tyrimas buvo atliktas siekiant įvertinti SARS-CoV-2 infekcijos seroepidemiologinį paplitimą tarp Lietuvos gyventojų.

**Medžiaga ir metodai.**

Tyrimas atliktas 2020 m. rugpjūčio–rugsėjo mėnesiais šešiose Lietuvos savivaldybėse. Imtį sudarė 3 087 suaugusieji iš bendrosios populiacijos (vidutinis amžius 53,7 m., 64 proc. moterys). SARS-CoV-2 antikūnai buvo įvertinti naudojant *AMP IgM/IgG Rapid Test*, kiti duomenys surinkti savistabos metodu. Seroepidemiologinis paplitimas nustatytas tiesiogiai vertinant testo rezultatus, taip pat juos koreguojant atsižvelgiant į testo jautrumą ir specifiškumą.

**Rezultatai.**

SARS-CoV-2 nekoreguotasis paplitimas visoje imtyje buvo 1,9 proc., koreguotasis – 1,4 proc. (nuo 0,8 iki 2,4 proc. atskirose savivaldybėse). Teigiamo testo atveju IgG buvo fiksuoti 67,2 proc., IgM – 29,3 proc., IgG ir IgM – 3,5 proc. Didesnę teigiamo testo tikimybę turėjo tyrimo dalyviai, kurie nurodė turėję kontaktą su artimos aplinkos žmonėms, kuriems buvo nustatyta SARS-CoV-2 infekcija (OR = 5,49, p < 0,001). Ribinis statistinis reikšmingumas nustatytas moterims (OR = 1,75, p = 0,082) ir nerūkantiesiems (OR = 2,95, p = 0,072). Iš seropozityvių tyrimo dalyvių 69,0 proc. nurodė neturėję COVID-19 simptomų po 2020 m. kovo 1 d., o 31,0 proc. teigė turėję bent vieną iš simptomų.

**Išvados.**

SARS-CoV-2 seroepidemiologinis paplitimas Lietuvos imtyje 2020 m. rugpjūčio–rugsėjo mėnesiais buvo 1,4 proc. (nuo 0,8 iki 2,4 proc. atskirose savivaldybėse). Vertinant oficialius duomenis, tyrimo pabaigoje (2020 m. rugsėjo 11 d.) Lietuvoje COVID-19 paplitimas nuo epidemijos pradžios siekė 117,5 atvejų 100 000 gyventojų, arba 0,12 proc. Tai leidžia spėti, kad viruso paplitimas populiacijoje buvo daugiau nei 10 kartų didesnis nei pagal oficialiąją statistiką.

**Raktažodžiai:** Seroepidemiologinis tyrimas; SARS-CoV-2; besimptomiai atvejai; Lietuva.

## Introduction

The novel betacoronavirus SARS-CoV-2 emerged in Wuhan (China) in 2019 and turned out to be very contagious [1] with presumably high mortality [2]. On 11 February 2020, the World Health Organization named the disease COVID-19, short for “coronavirus disease 2019” [3], and on 11 March 2020, the World Health Organization declared it a global pandemic [4]. During the first half of 2020, this disease spread rapidly all over the world resulting in many national lockdowns.

The first case of COVID-19 in Lithuania has been laboratory-confirmed on 28 February 2020 for a 39-year-old female who returned from a duty-travel in Verona (Italy). The national government has imposed strict lockdown measures on 16 March, when the total number of COVID-19 cases was 17 (0.61 per 100,000 population). The peak level of average 55 daily case-notification rate has been reached on 31 March, while the peak of active cases – 1047 (37.5 per 100,000) was registered on 19 April. Nonetheless, compared to other European countries, Lithuania has quite successfully controlled the first wave of the COVID-19 epidemic. By 30 June, there were relatively few confirmed cases and deaths – 1816 diagnosed cases (66.7 per 100,000) and 78 deaths (2.9 per 100,000) [5]. These rates were among the lowest ones across Europe.

However, results from other studies suggest that the real size of the pandemic is much higher than the officially confirmed numbers. The serological surveys are considered to be the best to define the spread of infectious disease, especially with asymptomatic cases [6]. Different studies from various countries have demonstrated that the seroprevalence of the SARS-CoV-2 virus is highly variable by region and time [7]. It is assumed that the data on the cumulative prevalence of this infection would help to understand the epidemiology of the outbreak [8].

Therefore, a national seroprevalence survey was carried out in Lithuania, using a random sample of the adult population. This was launched in August 2020 to estimate the real size of the COVID-19 epidemic in Lithuania and was aimed to establish the prevalence of seropositive persons in the general population and define the likely proportion of asymptomatic cases. We also wanted to analyze if the proportion of the seropositives varies by demographic characteristics.

## Materials and Methods

### Study design and procedures

The data of the study were collected from 10 August to 10 September 2020. The potential respondents received an invitation to participate in the study by filling in the questionnaire and giving the blood specimens for the serological test. The study sample was randomly selected from the Lithuanian State Enterprise Centre of Registers.

The study was conducted in three major cities (Vilnius, Kaunas, Klaipėda) and three selected municipalities (Tauragė district, Ukmergė district, and Zarasai district). The latter three municipalities were selected based on then-current COVID-19 morbidity indicators: Ukmergė district represented the highest level of morbidity, Tauragė district – medium, and Zarasai district – the lowest level. The required sample size was calculated using Raosoft calculator (http://www.raosoft.com/samplesize.html) with following assumptions: expected prevalence 3%, margin error 1%, confidence level 95%, population size – total population of municipalities.

Randomly selected adult participants (18 years and older) were asked by mail to visit a designated health care unit to undergo the SARS-CoV-2 antibodies test and fill in the questionnaire. The study participants have reported their age, gender, residence, education, health status, and COVID-19-related experience and behaviors. A seroconversion was evaluated using AMP IgM/IgG Rapid Test [9] from capillary blood, which was used for similar purposes in other studies [10]. This rapid immuno-chromatographic test can determine IgG and IgM antibodies separately as well as the combination of IgG/IgM antibodies against SARS-CoV-2. The capillary sampling was performed by the licensed specifically trained nurses. The total number of performed tests was 3087 (response rate 14.1%).

The study was approved by the Lithuanian Bioethics Committee on 8 July 2020, approval No. L-20-5/1. The respondents have signed the informed consent form.

### Statistical analysis

Univariate analysis and χ^2^ tests were used to analyze the relationships between the demographic and other characteristics of respondents and the prevalence of seropositives. Binary logistic regression was applied as univariate and multivariate analysis to test the relationship between the prevalence of seropositive respondents (who developed any type of antibodies or not) as the dependent variable, and characteristics used in the univariate analysis as the independent variables. A stepwise backward selection process of independent variables with p<0.30 was performed. Models were compared using the Akaike Information Criterion (AIC). Associations are presented in odds ratios (OR) with 95% confidence intervals (CI). They were considered statistically significant when p<0.05.

We estimated point estimate and CI of seroprevalence in six selected municipalities. Knowing that AMP IgM/IgG Rapid Test sensitivity is 92.0% and specificity 99.4% [11], we adjusted point estimates and CI to account for the validity of the test. The true seroprevalence was calculated following Rogan and Gladen [12]. The method for 95% exact CI for the true prevalence was proposed by Blaker [13]. The assumption about normal distribution was tested using the Shapiro-Francia normality test. The seroprevalence within subgroups was calculated with unadjusted (crude) scenarios.

The statistical analysis was conducted using statistical software of SPSS (IBM Corp. Released 2017. IBM SPSS Statistics for Windows, Version 25.0. Armonk, NY: IBM Corp.) and R (version 4.0.2).

## Results

The mean age of the study participants was 53.7 years (SD 16.6), median 55 years, range from 18 to 92 years. Based on the demographic profile it can be seen (Table 1) that almost half of the responders (45%) had a higher education (university or college), the majority were employed (60%). Looking into health profile it was found that most of the participants reported having no chronic conditions (61%) and were non-smokers (80%). To see the potential for contagion across international routes the respondents were asked if they have traveled abroad after 1 March 2020, when the pandemic was on its surge. In total, 16% of participants reported such trips starting this date onwards.

The study sample differed from the national population in that it had more people aged 50 years and more. Besides, the sample had an under-representation of men (36% compared to the national rate of 45%). The sample was selected to have an approximately similar number of participants from the selected municipalities, therefore in the study, they were more or less balanced (municipality’s share was between 12% and 22%), while in the national population the subgroups of municipalities were strongly dominated by the biggest cities of Vilnius, Kaunas, and Klaipėda.

The main outcome of this study was related to the seroprevalence rate. Overall, in the Lithuanian sample of selected municipalities, the crude seroprevalence of SARS-CoV-2 infection was 1.88% (95% CI 1.46–2.42%). Accounting for the externally validated sensitivity and specificity of the test, the seroprevalence was 1.40% (95% CI 0.92–1.99%) and ranged from 0.78% (95% CI 0.00–2.23%) to 2.44% (95% CI 1.02–4.49%) across different municipalities (Table 2). Among seroprevalent cases, two thirds of participants had IgG antibodies only (67.2%), 29.3% had IgM only, and 3.5% had both IgG and IgM.

**Table 1.** The study sample characteristics.

**Indicator**	**Sample (n=3087)**
**Age group**	
18–20 years	1.2% (36)
20–29 years	8.1% (251)
30–39 years	13.2% (408)
40–49 years	15.5% (477)
50–59 years	22.8% (703)
60–69 years	19.7% (609)
70–79 years	14.6% (450)
80+ years	4.9% (152)
**Gender**	
Males	36.1% (1114)
Females	63.9% (1973)
**Municipality**	
Vilnius city	17.3% (535)
Kaunas city	14.9% (460)
Klaipėda city	12.0% (370)
Ukmergė district	22.3% (689)
Tauragė district	18.7% (577)
Zarasai district	14.8% (456)
**Education**	n=3080
Basic and lower	5.9% (182)
Secondary	32.3% (995)
Vocational	16.4% (504)
Higher	45.4% (1399)
**Occupation**	n=3085
Students	2.7% (83)
Retired	27.3% (842)
Employed	59.5% (1835)
Unemployed, housewives, disabled	10.5% (325)
**Traveled abroad from 1 March 2020**	n=3086
Yes	16.3% (503)
No	83.7% (2583)
**Chronic diseases (at least one)**	n=3085
Yes	38.9% (1200)
No	61.1% (1885)
**Smoking status**	n=3085
Every day	15.0% (463)
Sometimes	5.0% (155)
No	79.9% (2467)

* Population information is retrieved from Statistics Lithuania, last update 28 May 2020.

**Table 2.** Seroprevalence by municipality

**Municipality**	**Target population (18+ years)**	**Tests performed**	**Seroprevalence (%, 95% CI)**	True seroprevalence* (%, exact 95% CI)	**Extrapolated seroprevalence in total population**	Officially confirmed cases as of PGR tests **
Vilnius city	454,707	535	1.31 (0.35–2.27)	0.78 (0.00–2.23)	1591–10322	931
Kaunas city	239,543	460	2.83 (1.31–4.35)	2.44 (1.02–4.49)	3136–10413	380
Klaipėda city	121,203	370	1.62 (0.33–2.91)	1.12 (0.12–3.08)	400–3527	345
Ukmergė district	28,303	689	1.45 (0.56–2.34)	0.93 (0.15–2.22)	158–662	93
Tauragė district	31,226	577	1.73 (0.67–2.79)	1.24 (0.31–2.77)	209–871	49
Zarasai district	12,722	456	2.63 (1.16–4.10)	2.22 (0.94–4.29)	148–522	5

* Seroprevalence is adjusted assuming sensitivity 92.0% and specificity 99.4%.

** By 12 September 2020.

Among the seropositive participants of the study, 69.0% reported having had no COVID-19 symptoms since 1 March 2020, while 31.0% reported having had at least one of the symptoms (fever, cough, muscle spasm, decrease or loss of taste, diarrhea, etc.).

Univariate analysis was conducted to see the subgroups of the population that are more likely to be diagnosed seropositive (Table 3). The results demonstrated significantly (p<0.05) higher seroprevalence among women (OR=1.51) and non-smokers (OR=3.37). Other subgroups having non-significant trends (0.05>p>0.30) for a higher proportion of seropositives were the elderly aged 65 years and more (OR=1.51), people above the basic education level, the employed people (OR=1.25), and participants within the normal BMI range compared to overweight and obese (OR=1.61).

The strongest and highly significant difference was detected among the people who reported close contact with other people who were diagnosed with SARS-CoV-2 infection (Table 3). Here it can be seen, that the former had 5.60 times higher odds for a seropositive test than the people who reported having had no contact with an infected person.

After the establishment of significant factors using univariate analysis, the multivariate regression was performed. The results showed (Table 4) that having close contacts with SARS-CoV-2 positives was the only significant factor associated with the status of being seropositive in our study. This factor was strong at the level of OR=5.49. The gender and smoking as factors were non-significant, but at the borderline significance (p<0.10).

**Table 3.** Seroprevalence by main characteristics: univariate logistic regression

**Indicator**	**Seropositive**	**OR (95% CI)**	**P**
**Age**			
18–64 years	1.38% (12/868)	1.00	
65+ years	2.07% (46/2218)	1.51 (0.78–3.15)	0.204
**Gender**			
Men	1.17% (13/1114)	1.00	
Women	2.28% (45/1973)	1.98 (1.04–4.01)	**0.029**
**Education**			
Basic or lower	0.55% (1/181)	1.00	
Secondary	1.84% (18/977)	3.33 (0.68–60.18)	0.242
Vocational	2.23% (11/493)	4.04 (0.78–74.09)	0.183
Higher	2.04% (28/1371)	3.70 (0.78–66.11)	0.200
**Smoking**
Daily	0.65% (3/463)	1.00	
Sometimes	1.29% (2/155)	2.00 (0.26–12.20)	0.449
Never	2.15% (53/2467)	3.37 (1.23–13.86)	**0.042**
**Employment**			
Not employed	1.63% (19/1167)	1.00	
Employed	2.03% (39/1918)	1.25 (0.70–2.31)	0.422
**Occupation**			
Students	1.20% (1/83)	1.00	
Retired	1.31% (11/842)	1.09 (0.21–19.97)	0.938
Employed	2.07% (38/1835)	1.73 (0.37 30.97)	0.589
Unemployed, housewives, disabled	2.46% (8/325)	2.07 (0.37–38.66)	0.496
**Chronic diseases (at least one)**			
Yes	1.67% (20/1200)	1.00	
No	2.02% (38/1885)	1.21 (0.68–2.21)	0.486
**Traveled abroad from 1 March 2020**			
No	1.82% (47/2583)	1.00
Yes	2.19% (11/503)	1.21 (0.56–2.38)	0.579
**BMI**			
18.5–24.99	2.46% (1/58)	1.61 (0.95–2.72)	0.075
<18.5	1.72% (28/1137)	1.12 (0.15–8.36)	0.912
25 and more	1.54% (29/1880)	1.00	
**Close contacts with SARS-CoV-2 positives**			
No	1.6% (46/2880)	1.00	
Yes	8.3% (8/96)	5.60 (2.56–12.22)	**<0.001**
Don’t know	3.7% (4/109)	2.35 (0.83–6.64)	0.108

**Table 4.** The risk factors for seropositivity: multivariate logistic regression

**Factor**	**Group**	**OR (95% CI)**	**P**
Gender	Men	1.00	
	Women	1.75 (0.93–3.28)	0.082
Smoking	Daily	1.00
	Sometimes	1.83 (0.30–11.15)	0.510
	Never	2.95 (0.91–9.58)	0.072
Close contacts with SARS-CoV-2 positives	No	1.00	
	Yes	5.49 (2.51–12.04)	<**0.001**
	Don’t know	2.31 (0.82–6.56)	0.115

## Discussion

The severe acute respiratory syndrome coronavirus 2 (SARS-CoV-2) was identified in December 2019. For this virus, it took less than three months to reach a pandemic level never seen for many decades. Since the first months of the spread of the virus, countries started the consistent monitoring of infection cases. As of 30 November 2020, there were more than 63 million COVID-19 cases worldwide, causing 1.47 million deaths and having 3% deaths among the closed cases [14]. However, the infection with SARS-CoV-2 can go asymptomatic, which means higher numbers of infection in populations – a recent meta-analysis found the proportion of asymptomatic cases being 17% [15]. For instance, in Spain this proportion varied from 22% to 36%, depending on the region [16]. Some researchers even suppose that the number of undiagnosed cases may be at least ten-fold higher than confirmed cases by PCR testing [7]. All this suggests the need for serological surveys to show the real spread of the virus within populations.

In our study, conducted in Lithuania during August–September 2020, we found that the seroprevalence in the total sample was 1.9%, ranging across municipalities from 1.3% to 2.8%. The sensitivity-specificity adjusted seroprevalence was 1.4%, ranging from 0.8% to 2.4%). A systematic review of seroprevalence studies by 1 May 2020 found that there were already 73 such studies [17]. The majority of them found seroprevalence higher than in our study, and not only in specific samples but in the general populations as well. The comparably low prevalence as in Lithuania was observed in several studies, such as 3% among blood donors in Paris and Oise region (France) [18], 2.8% among targeted Facebook users in Santa Clara County (California, US) [19] or 1.7% among blood donors in Denmark [20]. Similarly, the studies conducted at the population level in Europe showed seroprevalence at 5.0% in Spain [16] and 4.8% in Switzerland [21]. Many population-based studies found even larger prevalence rates, such as 6% in Miami (Florida, US), 6% in one town of Germany, or 21–33% in Iran [17]. There were also some studies where seroprevalence was very low, at 1% or even less [22].

We also assessed the antibody-specific profile of seroprevalence and found, that two thirds (67%) of seropositives had IgG antibodies, 29% had IgM, and 4% had both types of antibodies. These findings are comparable with other studies, such as among Croatian industry workers (53%, 32%, and 16%, respectively [9]).

However, in general it is hard to find consistency across different studies – the variation seems to have either a random-nature or can be explained by different external factors, related to the spread of infection as well as social and political environment. Interestingly, the findings of seroprevalence studies are as likely to be published in mass media as in research papers – probably due to the urgency of data and its potential for use in real-time and real-life situations.

The seroprevalence data is associated with the herd immunity and may be useful in predicting the effects on population mortality [23]. In the case of COVID-19, it is suggested that the herd immunity is likely to be reached if 60%, 70%, or even 80% of the population has recovered after infection, depending on the reproduction levels [24]. However, the herd immunity is hard to achieve, because high proportions of infection are related to high mortality in the susceptible population and the overburdening of health care systems [16].

In the Lithuanian seroprevalence study, we found that women have almost double the risk of a seropositive test – the absolute prevalence was 2.3% compared to 1.2% among men. This difference was statistically significant. However, it is hard to explain, because even though the men were under-represented in our sample, the gender ratio in our study compared to the general population was not that much different (36:64 and 45:55). Some studies show the findings suggesting higher seroprevalence among men but not women, for example, in South Korea [7] or California (the United States) [25]. However, an up-to-date review of surveys on SARS-CoV-2 antibody summed up that “seroprevalence does not differ significantly between males and females” [22].

Another very unexpected finding in our study was related to smoking – in univariate analysis, the data showed that non-smokers were at significantly higher risk of the seropositive test. The significance disappeared in multivariate analysis, however, it stayed at the borderline significance with an odds ratio close to 3. This association may have been due to other factors, for instance, the under-representation of smokers in our sample, where we had 20% of smokers, while in the total population of Lithuania this rate is 30% [26]. It may have happened that smokers with poorer health status or being unkeen to have health check-ups have decided not to enter our health-related study. The WHO update on COVID-19 and smoking has approached 26 studies and clearly showed the negative effects of smoking on COVID-19 [27]. However, even though smoking was associated with increased severity of COVID-19 and death in hospitalized COVID-19 patients, the evidence of the risk for infection was not yet available.

Serological surveys are important not only for demonstrating widespread and undiagnosed infection at the population level but also for the prognosis of epidemics. The detection of asymptomatic or subclinical infection of SARS-CoV-2 is essential for defining the extent and potential of the COVID-19 pandemic [8]. Therefore, serological testing for SARS-CoV-2 can supplement the efforts against this pandemic [28]. This is inevitable for implementing prevention measures along with planning and managing health care services. Seroprevalence data is particularly important in the current period when Lithuania is facing the second wave, which is considerably more pervasive as compared to the first spring wave. Moreover, the representative cross-sectional population studies on seroprevalence reveal the infection history [28].

In our study, we found the adjusted seroprevalence of 1.4%, which is more than 10 times higher than the officially reported national prevalence. This shows that seroprevalence studies are very relevant to estimate the real potential for COVID-19 morbidity and mortality since they are less biased [28]. The proportion of the population with SARS-CoV-2 cannot be assessed only on PCR confirmed cases. When conducting a seroprevalence survey in low-prevalence settings, to achieve better precision of point estimates of disease burden, the assay specificity should be prioritized, typically at the cost of sensitivity [28]. The seroprevalence data can be used to make political decisions related to the daily life of society – when to open or close schools, when to close down cultural events, and other relevant decisions.

It is relevant to discuss the potential limitations of our study. First, it is important to recognize the substantial differences in population size across the six municipalities in our survey. Therefore, one could argue that the primary results should be weighted according to population size. Although this is a reasonable argument, the very small number of positive cases in our survey renders the results very sensitive to the specific choice of weights. Therefore, we were in favor of presenting the seroprevalence adjusted for the validity of the test only.

Also, we cannot rule out the inaccuracy and reliability of the serology assay – the possibility of false-positive cases was already previously discussed [29]. Nevertheless, serological testing for antibodies IgM or IgG against SARS-CoV-2 is considered to be more accurate than the viral test, because antibodies are likely to stay for a longer time after viral infection [8].

Nonetheless, our study has also its strengths. The main strength is related to the sampling – we had a relatively large sample that was randomly selected from the general population. Quite many previous serological surveys were small or had specific subgroups such as health care staff or blood donors and therefore cannot provide the precise data on seroprevalence across the general population [16] or its demographic subgroups. On the other hand, we had an under-representation of the population aged under 50 years, which may have led to some imprecise estimates. Another strength is the serological test used in this study – it is established that AMP IgM/IgG Rapid Test had one of the highest sensitivities and specificities among the tests of its kind [30].

To finalize, we would like to overview the situation and management of the first COVID-19 wave in Lithuania. Our findings with low seroprevalence suggest that the first peak of COVID-19 outbreak (March–May 2020) was managed relatively successfully. We assume four main prerequisites for such an outcome:

1. The Lithuanian population has followed quarantine recommendations (keeping social distancing, wearing face masks, and disinfection) and reduced the mobility very significantly – retail and recreation by 72%, grocery and pharmacy by 38%, parks by 28%, and workplaces by 33% [31]. It is estimated that a mild quarantine regime such as one in Sweden could have risen a number of casualties up to 1094 by June 2020 [32].
2. The government has implemented very intensive testing strategy, using polymerase chain reaction (PCR) for detecting novel coronavirus. By 31 May 2020, Lithuania had performed 302,859 tests (or 11,125 per 100,000 population) and that was the 7^th^ largest number in the World [5].
3. Strict lockdown and intensive testing helped to flatten the curve of cases. The main COVID-19-related hospital care indicators are presented in Table 5. During the entire outbreak, the average of COVID-19 patients in all Lithuanian hospitals was 95.9 per day. This flow of patients was not a major challenge for health system, as Lithuania had 17,611 hospital beds, 651 beds in intensive care units, and 948 units for mechanical ventilation. Nevertheless, relatively high number of infected health care workers resulted in disturbances of the work in several hospitals.
4. Collaboration of different sectors: the COVID-19 outbreak encouraged active cooperation and networking among researchers, business, politicians, NGOs, health sector workers, and created more permanent networks.

**Table 5.** The utilization of hospital sector during the first COVID-19 outbreak in Lithuania

**Indicator**	**Weeks of 2020**											**Total**
	**14**	**15**	**16**	**17**	**18**	**19**	**20**	**21**	**22**	**23**	**24**	
Average number of hospitalized patients with COVID-19	115.8	133.8	135.9	140.3	116.6	96.0	95.9	84.7	66.4	52.4	33.6	96.5
Percentage of COVID-19 patients with oxygen mask	27.5	36.8	33.0	33.6	25.3	24.0	30.4	32.4	23.9	14.1	10.0	26.5
Percentage of COVID-19 patients with mechanical ventilation	2.7	5.9	6.7	7.5	7.0	7.8	4.6	2.6	2.8	2.0	3.9	4.9
Percentage of COVID-19 patients in intensive care units	6.1	9.7	11.3	12.7	13.4	13.6	11.2	8.7	7.3	5.7	9.0	10.1
Average number of COVID-19 infected physicians	55.8	53.3	37.6	28.1	21.4	13.1	13.3	12.3	14.1	11.0	9.0	22.8
Average number of COVID-19 infected nurses	50.8	68.3	68.6	59.7	50.7	38.6	39.0	33.7	31.3	30.1	29.9	45.1

Source: National Health Insurance Fund, Lithuania

## Conclusions

The results of the Lithuanian nationwide SARS-CoV-2 seroprevalence study were based on data from a representative sample of the population. The study showed that the seroprevalence by August–September 2020 was just 1.4%, ranging from 0.8% to 2.4% across municipalities. Two thirds of seropositive cases had IgG antibodies (67%), one third – IgM antibodies (29%), very rarely – both types (4%). By the end of our study, 11 September 2020 there was a total of 3199 cases of COVID-19 diagnosed in Lithuania, which was 117.5 per 100,000 population or 0.12%. In our study, we found the adjusted seroprevalence of 1.4%, which suggests more than 10 times higher prevalence of virus across the population than the official estimates.
